# Predicting standardized ileal digestibility of lysine in full-fat soybeans using chemical composition and physical characteristics

**DOI:** 10.5713/ab.23.0236

**Published:** 2024-01-20

**Authors:** Chanwit Kaewtapee, Rainer Mosenthin

**Affiliations:** 1 Department of Animal Science, Faculty of Agriculture, Kasetsart University, Bangkok 10900, Thailand; 2 University of Hohenheim, Institute of Animal Science, 70599 Stuttgart, Germany

**Keywords:** Digestibility, Heat Indicator, Lysine, Prediction, Soybean

## Abstract

**Objective:**

The present work was conducted to evaluate suitable variables and develop prediction equations using chemical composition and physical characteristics for estimating standardized ileal digestibility (SID) of lysine (Lys) in full-fat soybeans (FFSB).

**Methods:**

The chemical composition and physical characteristics were determined including trypsin inhibitor activity (TIA), urease activity (UA), protein solubility in 0.2% potassium hydroxide (KOH), protein dispersibility index (PDI), lysine to crude protein ratio (Lys:CP), reactive Lys:CP ratio, neutral detergent fiber, neutral detergent insoluble nitrogen (NDIN), acid detergent insoluble nitrogen (ADIN), acid detergent fiber, L* (lightness), and a* (redness). Pearson’s correlation (r) was computed, and the relationship between variables was determined by linear or quadratic regression. Stepwise multiple regression was performed to develop prediction equations for SID of Lys.

**Results:**

Negative correlations (p<0.01) between SID of Lys and protein quality indicators were observed for TIA (r = −0.80), PDI (r = −0.80), and UA (r = −0.76). The SID of Lys also showed a quadratic response (p<0.01) to UA, NDIN, TIA, L*, KOH, a* and Lys:CP. The best-fit model for predicting SID of Lys in FFSB included TIA, UA, NDIN, and ADIN, resulting in the highest coefficient of determination (R^2^ = 0.94).

**Conclusion:**

Quadratic regression with one variable indicated the high accuracy for UA, NDIN, TIA, and PDI. The multiple linear regression including TIA, UA, NDIN, and ADIN is an alternative model used to predict SID of Lys in FFSB to improve the accuracy. Therefore, multiple indicators are warranted to assess either insufficient or excessive heat treatment accurately, which can be employed by the feed industry as measures for quality control purposes to predict SID of Lys in FFSB.

## INTRODUCTION

Full-fat soybeans (FFSB) are an excellent source of protein and energy in pig diets [1]. However, the presence of heat-labile trypsin inhibitors in FFSB may cause a reduction in biological activity of trypsin, resulting in lower amino acid (AA) digestibility [2]. Currently, heat treatment of FFSB is being used to inactivate trypsin inhibitor activity (TIA) aiming to improve standardized ileal digestibility (SID) of crude protein (CP) and AA in piglets [2] and growing pigs [3]. However, excessive heat treatment may result in lower SID of AA in FFSB [3] due to destruction of chemical structure in AA and formation of Maillard reaction products [4]. Therefore, variation in protein quality of FFSB may be a result of either insufficient or excessive heat treatment.

The most commonly used indicators to assess differences in treatment of soybean products include urease activity (UA), protein solubility in 0.2% potassium hydroxide (KOH) and protein dispersibility index (PDI). These measurements are less expensive and more convenience in practice when compared with TIA [5]. Urease activity was in association with TIA as both enzymes were deactivated during heat treatment [5]. Alternatively, PDI and KOH can be used to reflect the solubility of protein fraction in response to the variation in heat processing [5]. Increasing heat treatment of soybean products showed lower ratios of lysine (Lys) to CP (Lys:CP) and reactive Lys (rLys) to CP (rLys:CP; [6]), and greater brown color [7]. Interestingly, increasing heat treatment also increased neutral detergent insoluble nitrogen (NDIN) content in FFSB [3], which may be used as an alternative variable to include in the prediction model for the extent of heat damage on SID of Lys. In the feed industry, there are several chemical and physical variables in place for quality control purposes. However, comparative studies aiming to assess the suitability of the individual variables including TIA, UA, PDI, KOH, Lys:CP, rLys:CP, neutral detergent fiber (NDF), NDIN, acid detergent insoluble nitrogen (ADIN), acid detergent fiber (ADF), and color values are lacking. The development of a prediction model can be used to evaluate soybean quality more efficiently, reducing time consumption. Thus, the objective of the present study was to evaluate suitable variables and develop prediction equations using chemical composition and physical characteristics for estimating SID of Lys of FFSB in growing pigs.

## MATERIALS AND METHODS

Data of previously published work [3] including information on SID of Lys, NDIN, TIA, UA, PDI, KOH, Lys:CP, rLys:CP, NDF, ADF, L*, and a* were used. The ratio of reactive Lys to Lys (rLys:Lys) and ADIN were used as the additional parameters to include in the model.

### Heat processing

**Figure 1:**
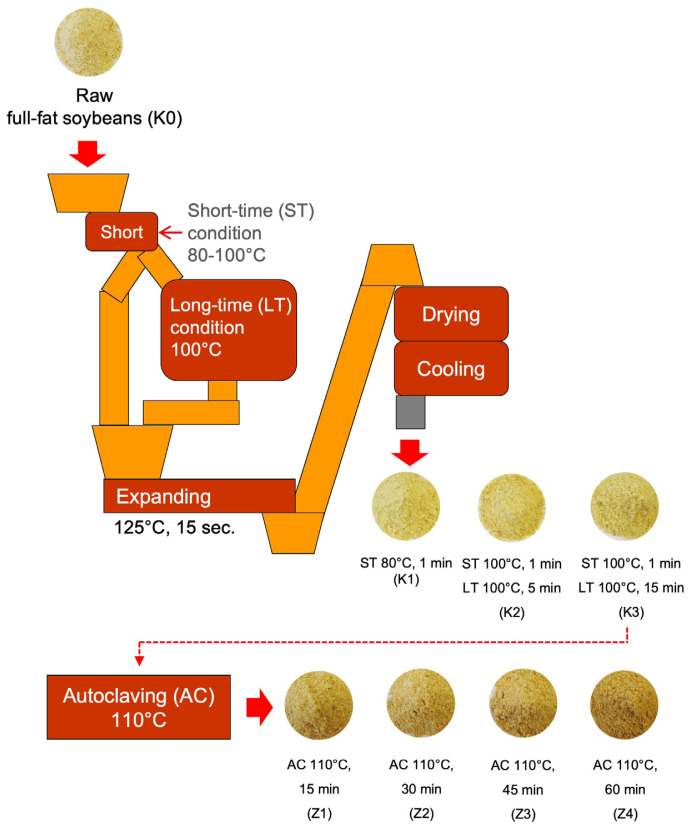
Flow chart of heat treatment of full-fat soybeans.

One batch of raw FFSB (K0) was mechanically broken and further processed using humidity conditions of wet heating procedure and autoclaving. The K0 was applied for short-time wet heating at 80°C for 1 min, followed by expansion at 125°C for 15 s to manufacture K1. Another 2 batches of K0 were processed using short-time wet heating at 100°C for 1 min, and thereafter long-time wet heating at 100°C for 5 min (K2) or 15 min (K3). These batches were expanded at 125°C for 15 s, followed by drying for 5 min and cooling for 5 min, respectively. The additional heat treatment of K3 was accomplished using autoclaving at 110°C for 15 (Z1), 30 (Z2), 45 (Z3), or 60 (Z4) min (Figure 1). In total, eight batches of FFSB with different heat treatment conditions were obtained from all of these processes. The chemical composition and soybean quality index of FFSB with different heat treatment conditions is shown in Table 1.

### Chemical analyses

Chemical composition of differently heated FFSB was analyzed according to official methods [8]. The NDIN and ADIN were analyzed as described by Haese et al [9]. The gas combustion (FP-2000, Leco Corp., St Joseph, MI, USA) was used to analyze nitrogen (N) content according to method 990.03 of the AOAC International [10]. Crude protein was calculated by multiplying the content of N by 6.25. Lysine content was determined using ion-exchange chromatography [11]. Reactive lysine was determined as outlined by Fontaine et al [6]. Briefly, 0.6 mol/L of O-methylisourea was used to react undamaged protein-bound lysine in FFSB to yield homoarginine. The condition was optimal at pH 11.5 for 48 h, followed by hydrolysis with 9 mol/L HCl for 23 h at 110°C. Homoarginine was determined using ion-exchange chromatography with postcolumn derivatization and then converted to rLys by the following equation:


rLys (%)=(homoarginine (%)/molecular weight of homoarginine)×molecular weight of Lys

where molecular weight of homoarginine is 188.23 g/mol and molecular weight of Lys is 146.19 g/mol. The TIA was analyzed following method 71-10 [12]. The KOH solubility was determined as described by Araba and Dale [13]. The determination of UA was based on the procedure of ISO 5506:1988 [14], whereas PDI was performed as outlined by AOCS [15]. The color of FFSB was measured as L* (lightness) and a* (redness) values using a Chroma Meter (Model CR-100; Minolta Camera Co., Ltd., Osaka, Japan).

### Statistical analysis

Pearson’s correlation (r) was computed using the CORR procedure of SAS (SAS Inst. Inc., Cary, NC, USA). Heatmap was created using library Seaborn [16] in python on Google Colab (Google Colaboratory, Mountain View, CA, USA). The relationship between SID of Lys and TIA, UA, KOH, PDI, Lys:CP, rLys:CP, rLys:Lys, crude fiber (CF), NDF, ADF, NDIN, ADIN, L*, and a* was determined by linear or quadratic regression using the general linear model (GLM) procedure of SAS. Stepwise multiple regression was performed to develop prediction equations for SID of Lys using the GLM procedure of SAS. Therefore, the prediction models included the following regression equations:


$$\begin{array}{l} \text{Linear regression: y}=\beta_{0}+\beta_{1}{\text{x}}_{1}\\ \text{Quadratic regression: y}=\beta_{0}+\beta_{1}{\text{x}}_{1}+\beta_{2}{{\text{x}}_{1}}^{2}\\ \text{Multiple linear regression: y}=\beta_{0}+\beta_{1}{\text{x}}_{1}+\beta_{2}{\text{x}}_{2}+\ldots +\beta_{\text{n}}{\text{x}}_{\text{n}} \end{array}$$

where y is the SID of Lys, β is a rate constant, and *x* is a variable. The R^2^ and root mean square error (RMSE) computed as


$${\text{R}}^{2}=1-\sqrt{\frac{\Sigma {\left({\text{y}}_{\text{i}}-\hat{\text{y}}\right)}^{2}}{\Sigma {\left({\text{y}}_{\text{i}}-\bar{\text{y}}\right)}^{2}}}\;\;\;\text{RMSE}=\sqrt{\frac{\Sigma ({\text{y}}_{\text{i}}-\hat{\text{y}})}{n}}$$

where y_i_ is the observed value, ŷ is the estimated value, ȳ is mean, and *n* is the number of observations. The high R^2^ and low RMSE were used as criteria for the most accurate model to predict SID of Lys. A p-value of <0.05 was considered significantly different.

## RESULTS

**Figure 2:**
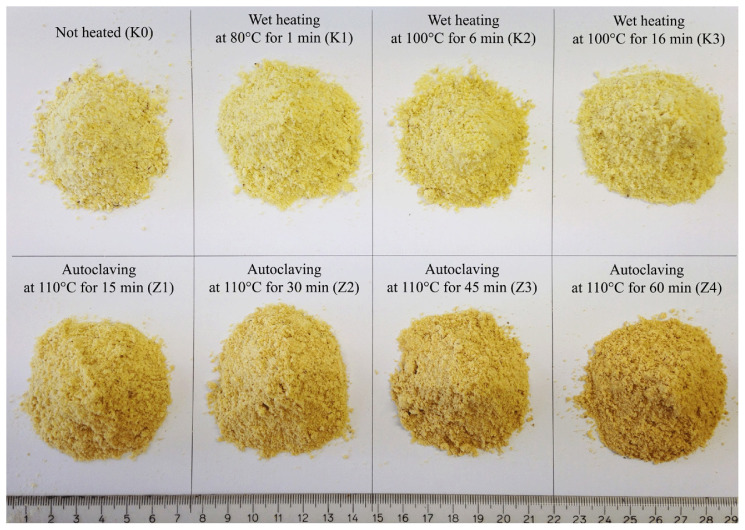
Full-fat soybeans at different heat treatment conditions.

**Figure 3:**
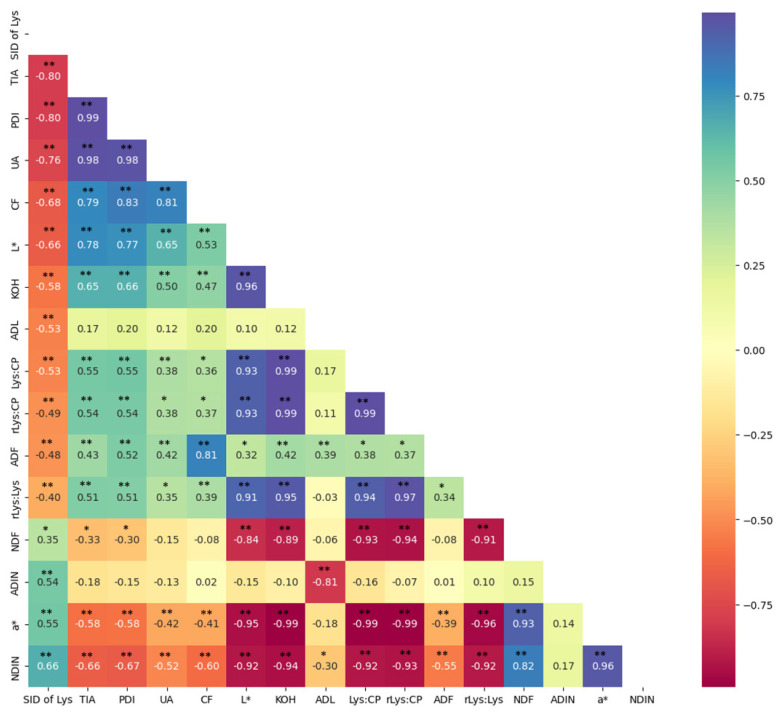
Pearson’s correlation between standardized ileal digestibility of lysine (SID of Lys) and chemical and physical characteristics of full-fat soybeans. ADF, acid detergent fiber; ADIN, acid detergent insoluble nitrogen; a*, redness; CF, crude fiber; KOH, protein solubility in 0.2% potassium hydroxide; Lys:CP, lysine to crude protein ratio; L*, lightness; NDF, neutral detergent fiber; NDIN, neutral detergent insoluble nitrogen; PDI, protein dispersibility index; rLys:CP, reactive lysine; rLys:Lys, reactive lysine to lysine ratio; TIA, trypsin inhibitor activity; UA, urease activity. Significances: * p<0.05, ** p<0.01.

The effect of heat processing on the change color of FFSB is displayed in Figure 2. Correlation analysis between SID of Lys and chemical composition and physical characteristics of FFSB is shown in Figure 3. High negative correlations (p<0.01) between SID of Lys and protein quality indicators were observed for TIA (r = −0.80), PDI (r = −0.80), and urease (r = −0.76). In addition, negative correlations (p<0.01) between SID Lys and chemical composition and physical characteristics were observed for CF (r = −0.68) and L* (r = −0.66). In contrast, positive correlations (p<0.01) between SID of Lys and chemical composition and physical characteristics for NDIN (r = 0.66), a* (r = 0.55), and ADIN (r = 0.54).

The equations for predicting SID of Lys from individual variables are shown in Table 2. The SID of Lys also showed a quadratic increase (p<0.01) to lower UA, TIA, KOH, Lys:CP, and L*, and greater NDIN, a*, and ADF. The SID of Lys linearly decreased (p<0.01) with greater PDI, CF, rLys:CP, and rLys:Lys, In addition, the SID of Lys linearly increased (p<0.01) with greater ADIN. The highest coefficient of determination (R^2^) and lowest RMSE were observed for UA.

An improvement in the precision of prediction equations can be obtained by using stepwise regressions (Table 3). The best-fit model included TIA, UA, NDIN, and ADIN, resulting in the highest coefficient of determination (R^2^ = 0.94) and the lowest error measurement (RMSE = 4.38) for predicting SID of Lys. Likewise, multiple linear regression including TIA, UA, NDIN, and ADIN increased the accuracy of the prediction model for most AA.

## DISCUSSION

The TIA in FFSB is responsible for negative effects on growth performance of pigs [17,18] due to the formation of inactive complexes with trypsin, chymotrypsin and other pancreatic enzymes [19]. Given this disadvantage, heat treatment has been used to reduce TIA in FFSB [20,21], which, in turn, resulted in higher SID of AA [3] and improved growth performance of pigs [22]. However, overheating may induce Maillard reaction products by increasing protein cross-links, which makes these products less soluble and less susceptible to digestive enzyme [23,24], thereby decreasing SID of AA in soybean products [7,25]. Results of the present study are in agreement with previous data, where high negative correlation was observed between SID of Lys and TIA. Furthermore, increasing heat treatment showed a quadratic response of greater SID of Lys to lower TIA. Notably, the lowest TIA in FFSB did not correspond to highest SID of Lys due to excessive heat damage, which was indicated by lower accuracy of TIA (R^2^ = 0.76) when compared to UA (R^2^ = 0.86) and NDIN (R^2^ = 0.82).

In the feed industry, several chemical and physical methods including determination of UA, KOH, and PDI are widely used to determine the degree of heat damage of soybean products [5,25]. In the present study, SID of Lys showed a quadratic response to decreasing UA and KOH, and a linear decrease with increasing PDI. Urease activity had the higher R^2^ (0.86) with lower RMSE (6.45) compared to PDI (R^2^ = 0.71, RMSE = 9.05) and KOH (R^2^ = 0.68, RMSE = 9.71). It has to be emphasized that the observed decrease in UA was associated with a similar decline in TIA with increasing heat treatment [26], whereas low PDI and KOH values reflect the lower solubility of the protein fraction in response to the high heat processing [5,27]. Under practical conditions, the TIA analysis is more time-consuming and expensive in comparison to UA, KOH, and PDI determination. Among these parameters based on R^2^ and RMSE obtained in the present study, determining UA or PDI is cost effective approaches for predicting SID of Lys with high accuracy.

Excessive heat treatment may induce the ɛ-amino group to react with reducing sugars [28,29]. Among AA, Lys is most sensitive to heat damage, consequently, the destruction of Lys is reflected in lower Lys:CP and rLys:CP [6]. In the present study, the SID of Lys showed a quadratic response with decreasing Lys:CP, and a linear increase with decreasing rLys:CP and rLys:Lys. Furthermore, the reducing sugars in fiber fractions represent the most reactive carbohydrate fraction during Maillard reaction [30]. According to Pastuszewska et al [31] and Eklund et al [32], prolongation of heat treatment of rapeseed products resulted in higher NDF and NDIN contents due to the formation of Maillard reaction products between protein and NDF during the intensify of heat treatment. In the present study, the SID of Lys showed a linear increase with increasing NDF, and a quadratic response with increasing NDIN. Increasing heat treatment of raw FFSB resulted in higher NDF and NDIN contents, whereas the SID of Lys increased from the under-heating to optimal heat processing condition due to the lower TIA. Thereafter, further heat treatment resulted in lower SID of Lys due to the greater formation of Maillard reaction products. Considering all the parameters within this group, the reliability of NDIN as a variable for predicting the SID of Lys in heat-treated protein supplements is notable due to the high accuracy of the prediction model.

The development of brown color in FFSB has been proposed to indicate the presence of Maillard reaction products [6]. These changes in color have been reported to coincide with changes in SID of Lys in soybean meal [7] and canola meal [33]. For example, a decrease in SID of CP and AA has been associated with darker and redder colors (lower L* and greater a* values) due to a greater presence of Maillard reaction products [7]. This is in accordance with the results of the present study, where SID of Lys showed a quadratic response to lower L* and greater a* values. Compared to chemical analyses of variables, color measurements represent a rapid method for predicting SID of Lys in pigs.

In comparison to individual variables, multiple linear regression including TIA, UA, NDIN and ADIN increased the accuracy (R^2^ = 0.93 to 0.96) of the prediction model with low error measurement (RMSE = 3.96 to 5.35) for most AA. Possibly, TIA represents the anti-nutritional factors in soybean products [17]. Furthermore, UA in soybean products will be completely destroyed, thus, there is no further response to additional heat treatment. On the other hand, prolonged heat treatment leads to increased contents of NDIN. Consequently, NDIN serve as reliable indicators of the extent of heat treatment, while UA primarily reflects the presence of anti-nutritional factors, similar to TIA. Therefore, a multiple linear regression including TIA, UA, NDIN, and ADIN was the best fit model for predicting SID of most AA in soybean products.

## CONCLUSION

The TIA, PDI, and UA were suitable common indicators of soybean quality related to SID of Lys due to high negative correlation. For the prediction model, the quadratic regression with one variable indicated the four highest coefficients of determination for UA (R^2^ = 0.86), NDIN (R^2^ = 0.82), TIA (R^2^ = 0.76), and PDI (R^2^ = 0.71). The multiple linear regression including several variables is an alternative model used to predict SID of all AA in FFSB. In conclusion, multiple indicators are warranted to assess either insufficient or excessive heat treatment accurately, which can be employed as measures for quality control purposes to predict SID of Lys in FFSB.

## Figures and Tables

**Table 1:** Chemical composition and soybean quality index of full-fat soybeans with different heat treatment conditions

Item	Raw FFSB	Heat processing						

		Short-time condition			Short-time condition			
	
		80°C	100°C	100°C	100°C			
	
		1 min	1 min	1 min	1 min			
	
			Long-time condition, 100°C		Long-time condition, 100°C			
	
			5 min	15 min	15 min			
	
		Expanding, 125°C 5 s			Expanding, 125°C 5 s			

					Autoclaving, 110°C			

					15 min	30 min	45 min	60 min
	
	(K0)	(K1)	(K2)	(K3)	(Z1)	(Z2)	(Z3)	(Z4)
SID of Lys (%)	47.56	57.44	78.04	83.97	83.76	83.88	84.96	82.03
Chemical composition (% DM)								
CP	39.23	39.41	39.41	39.45	39.81	40.15	39.71	40.22
Lys	2.45	2.47	2.46	2.46	2.43	2.38	2.33	2.32
rLys	2.26	2.28	2.28	2.28	2.21	2.16	2.09	2.05
Lys:CP	6.24	6.28	6.24	6.24	6.11	5.92	5.86	5.77
rLys:CP	5.76	5.78	5.78	5.79	5.56	5.38	5.27	5.10
rLys:Lys	92.36	92.09	92.56	92.75	90.99	90.92	89.94	88.42
CF	4.95	4.06	4.43	3.46	3.65	3.96	4.02	3.57
NDF	10.76	8.97	10.65	8.65	10.82	11.95	13.09	14.37
NDIN	0.10	0.10	0.20	0.40	0.70	0.60	0.90	1.10
ADF	6.31	6.08	6.89	5.13	5.61	5.71	5.70	5.56
ADIN	0.08	0.05	0.10	0.09	0.09	0.10	0.09	0.07
Soybean quality index								
TIA (TIU/g)	29.49	7.75	6.36	5.73	2.59	1.62	1.07	0.86
UA (mg N_2_/g min)	4.48	0.45	0.39	0.05	0.02	0.01	0.00	0.00
PDI (%)	72.20	22.00	24.60	14.70	9.80	7.50	7.00	6.80
KOH (%)	94.20	90.00	89.50	88.70	81.40	71.80	66.90	60.30
L*	65.27	62.34	60.68	62.42	59.38	57.97	56.54	54.56
a*	−0.88	−1.20	−0.57	−0.69	1.98	3.29	4.59	6.17

FFSB, full-fat soybeans; SID of Lys, standardized ileal digestibility of lysine; CP, crude protein; Lys, lysine; rLys, reactive lysine; Lys:CP, lysine to crude protein ratio; rLys:CP, reactive lysine to crude protein ratio; rLys:Lys, reactive lysine to lysine ratio; CF, crude fiber; NDF, neutral detergent fiber; NDIN, neutral detergent insoluble nitrogen; ADF, acid detergent fiber; ADIN, acid detergent insoluble nitrogen; TIA, trypsin inhibitor activity; TIU, trypsin inhibitor units; UA, urease activity; PDI, protein dispersibility index; KOH, protein solubility in 0.2% potassium hydroxide; L*, lightness; a*, redness.

**Table 2:** Linear and quadratic regression equations for standardized ileal digestibility of lysine (SID of Lys) from single variable in full-fat soybeans1)

No	Variable	Equation	R^2^	RMSE	p-value	

					Linear	Quadratic
1.	UA	SID of Lys = 84.64−46.94(UA)+8.63(UA^2^)	0.86	6.45	<0.001	<0.001
2.	NDIN	SID of Lys = 47.78+107.14(NDIN)−71.22(NDIN)^2^	0.82	7.38	<0.001	<0.001
3.	TIA	SID of Lys = 88.45−2.79(TIA)+0.05(TIA)^2^	0.76	8.35	<0.001	<0.001
4.	PDI	SID of Lys = 86.91−0.58(PDI)	0.71	9.05	0.001	0.060
5.	L*	SID of Lys = −1,776.72+65.12(L*)−0.57(L*)^2^	0.70	9.36	<0.001	<0.001
6.	KOH	SID of Lys = −296.25+10.63(KOH)−0.07(KOH)^2^	0.68	9.71	<0.001	<0.001
7.	a*	SID of Lys = 73.46+7.46(a*)−1.03(a*)^2^	0.54	11.60	<0.001	0.003
8.	CF	SID 0f Lys = 162.03−21.66(CF)	0.53	11.55	<0.001	0.055
9.	Lys:CP	SID of Lys = −8,053.60+2,735.68(Lys:CP) −229.82(Lys:CP)^2^	0.50	12.19	0.008	0.007
10.	ADF	SID of Lys = 738.22−208.56(ADF)+16.18(ADF)^2^	0.40	13.25	0.007	0.011
11.	ADIN	SID of Lys = 35.24+485.26(ADIN)	0.37	13.47	<0.001	0.805
12.	rLys:CP	SID of Lys = 224.49−26.83(rLys:CP)	0.33	13.85	0.001	0.086
13.	rLys:Lys	SID of Lys = 440.85−4.00(rLys:Lys)	0.25	14.68	0.006	0.444
14.	NDF	SID of Lys = 46.10+2.65(NDF)	0.20	15.10	0.018	0.573

RMSE, root mean square error; UA, urease activity; NDIN, neutral detergent insoluble nitrogen; TIA, trypsin inhibitor activity; PDI, protein dispersibility index; L*, lightness; KOH, protein solubility in 0.2% potassium hydroxide; a*, redness; a*, redness; CF, crude fiber; Lys:CP, lysine to crude protein ratio; ADF, acid detergent fiber; ADIN, acid detergent insoluble nitrogen; rLys:CP, reactive lysine to crude protein ratio; rLys:Lys, reactive lysine to lysine ratio; NDF, neutral detergent fiber.

1) There are 46 observations for the linear and quadratic regression equations.

**Table 3:** Stepwise regression equations for standardized ileal digestibility (SID) of amino acids

Item	Equation	R^2^	RMSE
Indispensable amino acids			
Arginine (Arg)	SID of Arg = 64.71−5.61(CF)+16.33(NDIN)−3.41(UA)+394.26(ADIN)	0.95	4.17
Histidine (His)	SID of His = 23.30+2.23(TIA)+26.81(NDIN)−16.52(UA)+413.33(ADIN)	0.94	4.19
Isoleucine (Ile)	SID of Ile = 55.75−7.53(CF)+19.83(NDIN)−3.90(UA)+476.27(ADIN)	0.96	4.36
Leucine (Leu)	SID of Leu = 7.31+2.60(TIA)+35.94(NDIN)−19.24(UA)+502.12(ADIN)	0.96	4.34
Lysine (Lys)	SID of Lys = 23.91+2.47(TIA)−18.85(UA)+24.55(NDIN)+409.67(ADIN)	0.94	4.38
Methionine (Met)	SID of Met = 17.05+2.42(TIA)+31.66(NDIN) −18.10(UA)+442.55(ADIN)	0.94	4.86
Phenylalanine (Phe)	SID of Phe = 7.97+2.44(TIA)+34.23(NDIN)−18.60(UA)+507.24(ADIN)	0.96	4.41
Threonine (Thr)	SID of Thr = 29.06−2.46(a*)−0.32(PDI)+34.05(NDIN)+388.86(ADIN)	0.94	4.51
Tryptophan (Trp)	SID of Trp = 60.71−9.29(CF)−24(PDI)+17.25(NDIN)+501.81(ADIN)	0.96	4.27
Valine (Val)	SID of Val = 13.00+2.57(TIA)+32.65(NDIN)−18.67(UA)+446.10(ADIN)	0.96	3.96
Dispensable amino acids			
Alanine (Ala)	SID of Ala = 34.87−2.28(a*)−0.33(PDI)+32.18(NDIN)+366.66(ADIN)	0.95	4.18
Aspartic acid (Asp)	SID of Asp = 81.22−11.60(CF) −0.63(TIA)+483.95(ADIN)	0.93	4.75
Cystine (Cys)	SID of Cys = 74.94−13.07(CF)−0.69(TIA)+550.51(ADIN)	0.93	5.35
Glutamic acid (Glu)	SID of Glu = 26.26+1.87(TIA)+24.75(NDIN)−14.60(UA)+403.01(ADIN)	0.93	4.82
Glycine (Gly)	SID of Gly = 30.89−2.55(a*)−0.32(PDI)+34.05(NDIN)+382.48(ADIN)	0.94	4.52
Proline (Pro)	SID of Pro = 23.79+2.12(TIA)+28.98(NDIN)−16.27(UA)+496.40(ADIN)	0.93	5.28
Serine (Ser)	SID of Ser = 5.36+2.77(TIA)+34.47(NDIN)−20.33(UA)+508.92(ADIN)	0.96	4.49

RMSE, root mean square error; CF, crude fiber; NDIN, neutral detergent insoluble nitrogen; UA, urease activity; ADIN, acid detergent insoluble nitrogen; ADL, acid detergent lignin; TIA, trypsin inhibitor activity; a*, redness; PDI, protein dispersibility index.
